# Correlation between macular edema recurrence and macular capillary network destruction in branch retinal vein occlusion

**DOI:** 10.1186/s12886-020-01611-w

**Published:** 2020-08-24

**Authors:** Ji Hye Jang, Yu Cheol Kim, Jae Pil Shin

**Affiliations:** 1 Department of Ophthalmology, Keimyung Universtiy School of Medicine, Daegu, Republic of Korea; 2 Keimyung University Institute for Medical Science, Daegu, Republic of Korea; 3 Department of Ophthalmology, Kyungpook National University School of Medicine, Daegu, Republic of Korea

**Keywords:** Branch retinal vein occlusion, Macular capillary network, Macular edema, Optical coherent tomography angiography, Vessel density

## Abstract

**Background:**

The aim of this study was to evaluate the correlation between changes in the macular capillary network and macular edema (ME) recurrence with branch retinal vein occlusion (BRVO) using swept-source optical coherence tomography angiography (SS-OCTA).

**Methods:**

We reviewed the data for 43 patients with treatment-naïve ME associated with BRVO. Patients who received intravitreal bevacizumab injection were divided into two groups based on ME recurrence at 6 months after edema resolution. The perifoveal capillary morphology and the macular capillary vessel density (VD) were retrospectively analyzed using *en face* SS-OCTA after ME resolution.

**Results:**

The perifoveal capillary ring loss in the superficial capillary plexus (SCP) and deep capillary plexus (DCP) was more common in the ME recurrence group (*n* = 22) than in the no ME recurrence group (*p* = 0.047 and *p* = 0.002). Relative to the findings in the no ME recurrence groups, the destruction of the perifoveal capillary ring was more severe in the DCP (30.0° vs 87.3°, *p* = 0.001) than in the SCP (17.3° vs 69.5°, *p* = 0.006) in the ME recurrence group. The hemi-VD disparity between the affected and the unaffected areas in the SCP and DCP showed significant differences (*p* = 0.031 and *p* = 0.017), while macular VD showed no differences between the groups.

**Conclusions:**

Destruction of the perifoveal capillary ring and hemi-VD disparity could be related to ME recurrence in BRVO. Therefore, these factors may be helpful in predicting ME recurrence.

## Background

Macular edema (ME) is the most common cause of visual loss in patients with branch retinal vein occlusion (BRVO). It is caused by physical destruction of the inner blood-retinal barrier due to elevated venous pressure as a result of vein occlusion at the arteriovenous crossing site [1, 2]. With respect to the natural progression of BRVO, ME occurs in 5–15% of the cases within 1 year from the initial onset. While spontaneous resolution is achieved in less than half the cases, recovery of visual acuity to 20/40 or better is rare [3].

Other factors involved in the onset of ME include increased vascular permeability caused by vascular endothelial growth factors (VEGFs) or various inflammatory cytokines [4, 5]. Increased levels of intravitreal VEGFs are associated with nonperfusion areas in the retinal capillaries and the severity of ME [5, 6]. However, many cases still require re-treatment because of recurrence or persistence of ME despite intravitreal anti-VEGF and/or steroid injection therapy [7–9]. Yoo et al. [10] reported that recurrent ME requires more aggressive treatment and that spontaneous resolution without any treatment is very rare.

According to Spaide’s [11] new theory, ME related to retinal vascular disorders is associated with regulation of the flow of fluid in macular capillaries. Edema in BRVO primarily occurs in the same areas of altered macular capillary flow [11]. Tsuboi et al. [12] reported that persistent macular edema can be related to the difference in capillary loss between the deep capillary plexus (DCP) and the superficial capillary plexus (SCP). However, data on the relationship between changes in the macular capillary network and ME recurrence are lacking.

Optical coherence tomography (OCT) is a very important diagnostic tool for ME caused by RVO [13, 14]. It takes images of cross-sections of the macular region and provides information about changes in the macular thickness, changes in intraretinal cysts, accumulation of subretinal fluid, and photoreceptor damage. However, OCT cannot show changes in the foveal avascular zone (FAZ) or the different layers of the capillary network.

In contrast, OCT angiography (OCTA) without a contrast agent uses light with a wavelength of 840–1050 nm, to amplify and calculate the differences in the signals emitted from moving and non-moving tissues, and detect the flow of erythrocytes within the blood vessels, thereby producing reconstructed images of the retinal and choroidal vascular structure. The data obtained from the retinal volume scan are reconstructed as *en face* images, and they also provide information about the capillary morphology and FAZ area [15–17]. Therefore, in the present study, we used SS-OCTA to analyze changes in the macular capillary structure following treatment for BRVO-induced ME and investigated the factors associated with ME recurrence.

## Methods

The present study retrospectively analyzed the medical records and images of patients diagnosed with treatment-naïve ME associated with BRVO at the Department of Ophthalmology, Dongsan Medical Center, Keimyung University between October 2016 and March 2018. Comparative analysis was performed with the patients divided into two groups based on ME recurrence (ME recurrence and no ME recurrence) during 6 months after the resolution of initial ME. The present study was performed in accordance with the principles of the Declaration of Helsinki and approved by the Keimyung University Institutional Review Board (IRB no. 2018–09-039).

Patients with any of the following conditions were excluded from the study: 1) previous diagnosis and treatment for BRVO; 2) other retinal diseases that can affect the macular thickness, such as age-related macular degeneration, diabetic retinopathy, central retinal vein occlusion, and epi-retinal membrane; 3) high myopia (axial length ≥ 26.5 mm or refractive error ≥ − 6 diopter); 4) glaucoma; and 5) history of pars plana vitrectomy. Patients were also excluded in case of voluntary termination of follow-up prior to ME resolution and difficulty in data analysis due to poor image quality.

The locations of vein occlusion proposed by Hayreh et al. [18] were used to divide the occlusion into two types: major BRVO and macular BRVO. ME was diagnosed using swept source-OCT (SS-OCT; Swept Source DRI-OCT Triton™, Topcon, Tokyo, Japan) and macular thickening was defined as a central macular thickness (CMT) of ≥300 um with intraretinal cysts or subretinal fluid in the macular region. OCT was performed during each visit to check for the presence of edema and changes in the macular thickness. Resolution of ME was defined as a CMT of < 300 um with a concave macular contour. All patients were treated with intravitreal bevacizumab injections for ME. During the follow-up periods, intravitreal bevacizumab injection were repeated as needed until ME resolution was achieved.

### Analysis of the perifoveal capillary network morphology using OCTA

Changes in the morphology of the perifoveal capillary network (FAZ area, perifoveal capillary ring) were analyzed using SS-OCTA (Swept Source DRI-OCT Triton™, Topcon, Tokyo, Japan) and imaging was performed by a single experienced examiner on the same day.

The *en face* images of the macular region (3 × 3 mm) were automatically acquired with four slabs divided into the SCP, DCP, outer retina, and choriocapillaris using the IMAGEnet 6 software (version 1.17, Topcon, Tokyo, Japan). The SCP included the area from a point 2.6 um below the internal limiting membrane to a point 15.6 um below the inner plexiform layer, while the DCP included the area between 15.6 um to 70.2 um below the inner plexiform layer.

For the elimination of segmentation errors in the retinal layer due to macular swelling, analyzed images were obtained after resolution of ME. Images with signal strength intensity (SSI) values of ≥50, and with the centrally located fovea were selected, and the FAZ area and perifoveal capillary ring morphology were analyzed.

**Fig. 1:**
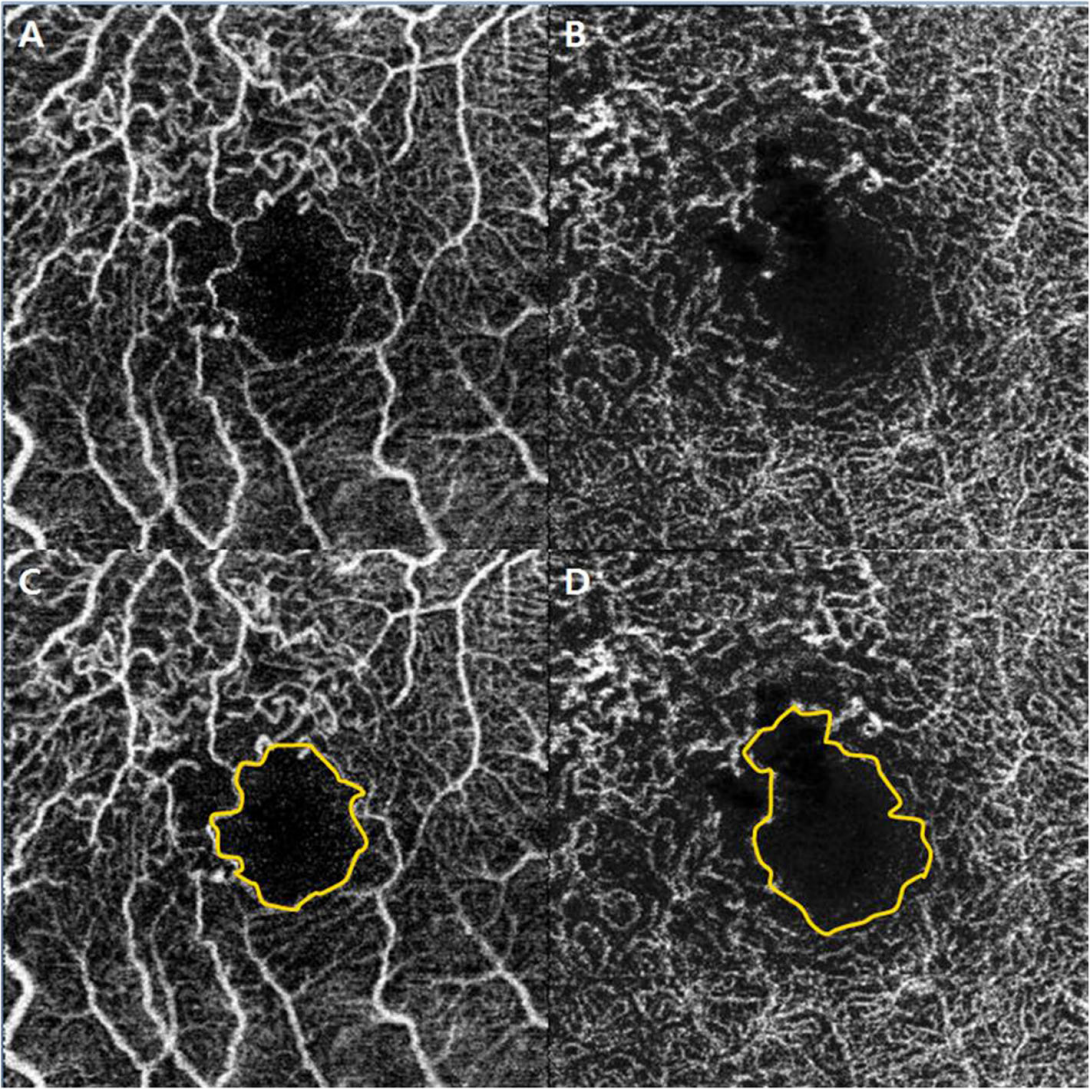
Macular capillary network parameter on e*n face* 3 × 3-mm swept-source OCTA. The foveal avascular zone area in the superficial capillary plexus (**a**) and deep capillary plexus (**b**) is automatically measured if it is manually drawn along the inner boundary of the capillary network (**c** and **d**). OCTA: optical coherence tomography angiography

The FAZ, defined as the avascular area in the center of the fovea was manually outlined by two retinal specialist independently (JHJ and YCK), using a caliper contained in the program (Fig. 1). The FAZ areas of the SCP and DCP were calculated using the built- in program that measures the outlined area. A mean value of two measurements was used for the analysis.

**Fig. 2:**
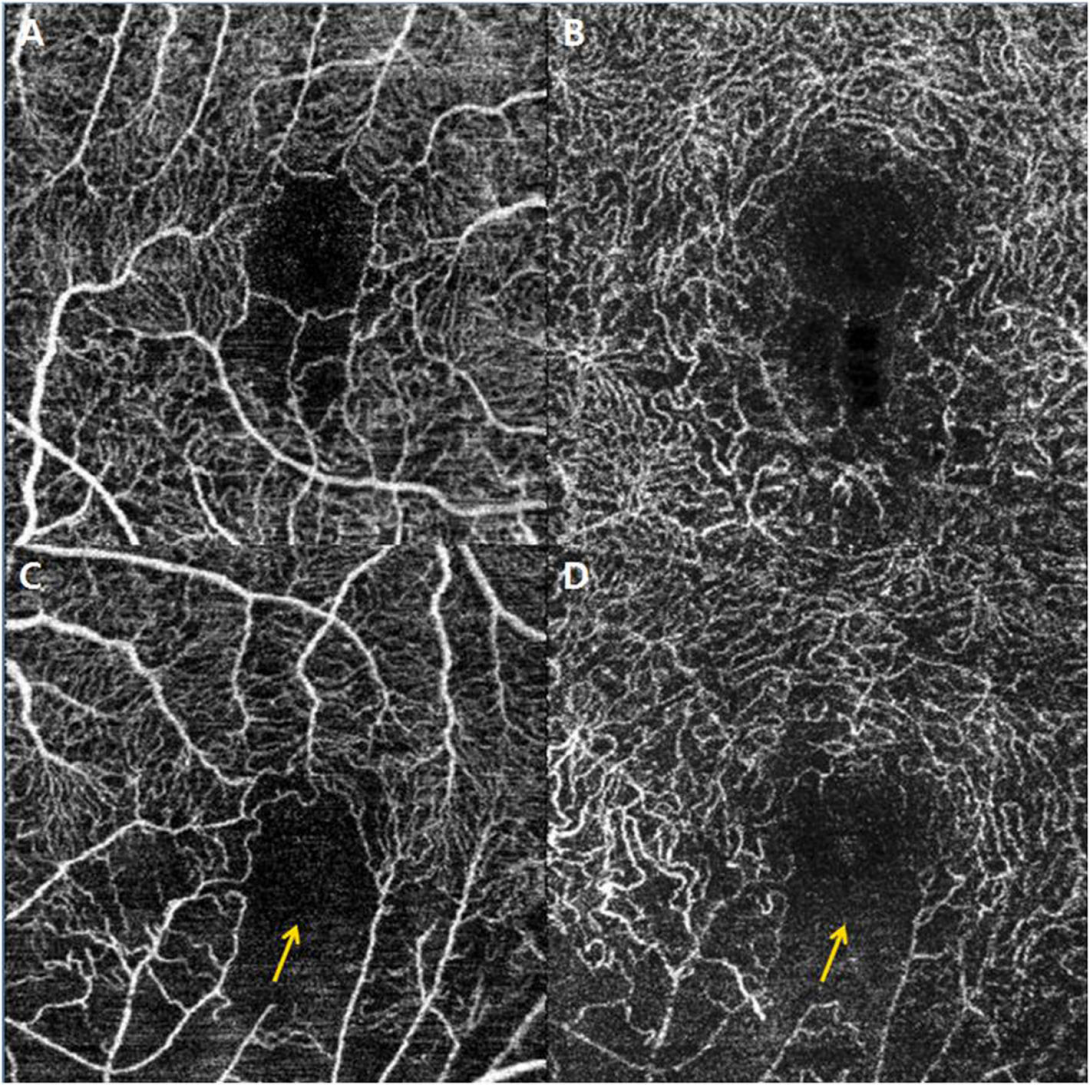
Perifoval capillary ring morphology after macular edema resolution on *en face* 3 × 3-mm swept-source OCTA. (**a** and **b**) These images show slight perifoveal capillary loss with an intact capillary ring in the superficial capillary plexus and deep capillary plexus. **c** and **d** These images show disruptions of the perifoveal capillary ring in the superficial capillary plexus and deep capillary plexus. OCTA: optical coherence tomography angiography

The perifoveal capillary ring was defined as the inner boundary of FAZ. Changes in the perifoveal capillary ring were divided into two types: (I) intact (0 clock hour) if the inner boundary of the capillary plexus was not broken after resolution of ME, and (II) ring loss or destruction if the inner boundary was broken. The degree of ring loss was marked as the clock hour and converted by multiplying each hour by 30° (Fig. 2).

### Analysis of macular capillary VD using ImageJ

**Fig. 3:**
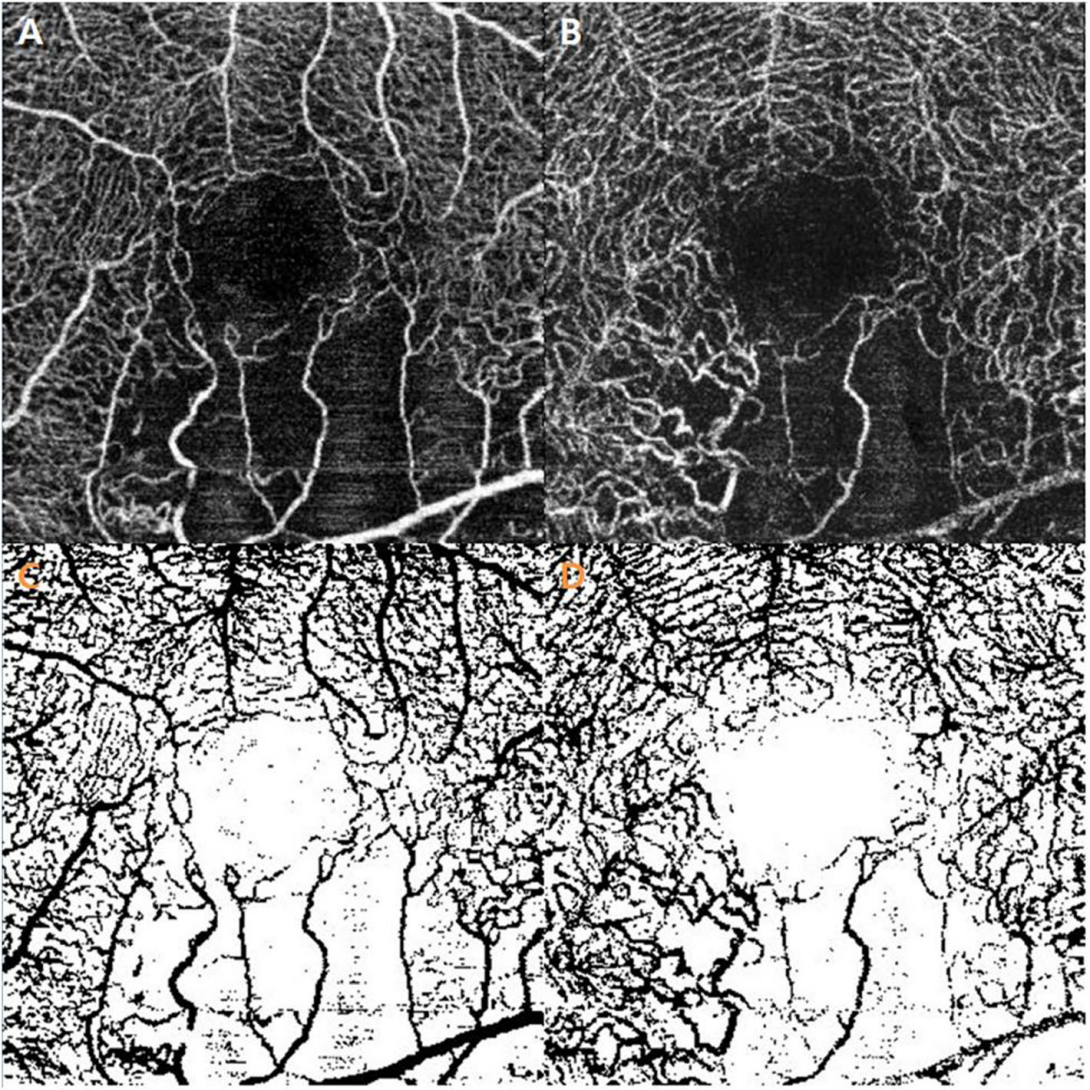
Macular vessel density assessment using the binarization process. The ImageJ program of *en face* 3 × 3-mm swept-source OCTA was used. **a** and **b** These images show large capillary loss in the inferior region of the superficial capillary plexus and deep capillary plexus. **c** and **d** The vessel density is calculated in the 320 × 320-pixel area on binarized images of the superficial capillary plexus and deep capillary plexus. OCTA: optical coherence tomography angiography

VD in the macular capillary network was analyzed by the ImageJ software program (version 1.52a, National Institutes of Health, Bethesda, Maryland, USA; available at http://imagej.nih.gov/ij/) using SCP and DCP images acquired by OCTA for 3 × 3 mm macular region. For visualization of the capillaries, a 320 × 320-pixel image was converted to an 8-bit image and processed by binarization after marking with gray values between 0 and 255, and the average gray value of all pixels was used as the VD (Fig. 3).

**Fig. 4:**
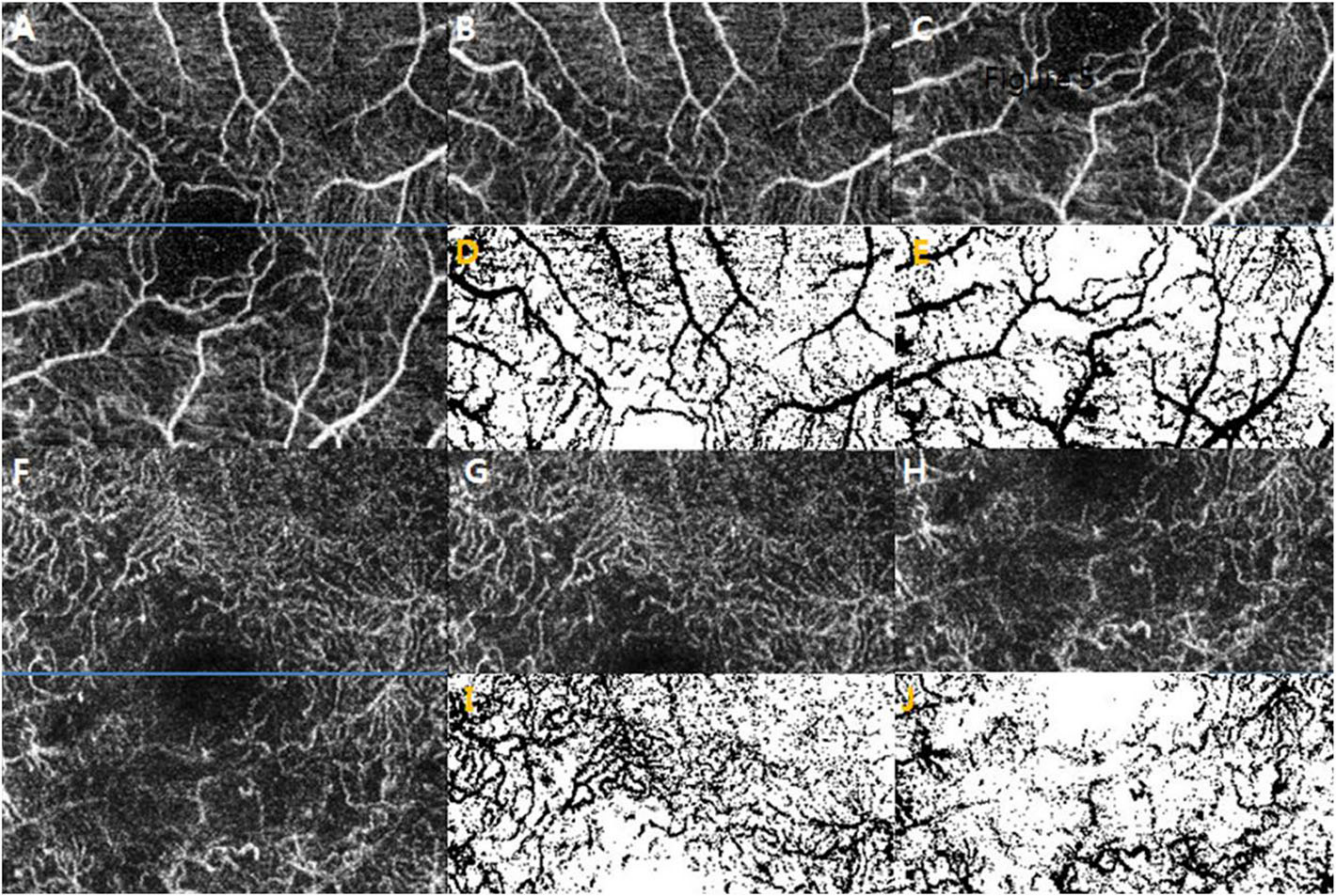
Hemi-vessel density disparity in the SCP and DCP. An *en face* 3 × 3-mm OCTA image of the SCP (**a**) is divided into two regions, a hemi-superior macular area (**b**) and a hemi-inferior macular area (**c**) by the horizontal blue line. The hemi-vessel density disparity in the SCP is derived by subtracting the vessel density values of the affected hemi-area from the unaffected hemi-area obtained from the binarized images (**d**, **e**). An *en face* 3 × 3-mm OCTA image of the DCP (**f**) is divided into a hemi-superior macular area (**g**) and a hemi-inferior macular area (**h**) by the blue line. The hemi-vessel density disparity in the DCP is derived by subtracting the vessel density values of the affected hemi-area from the unaffected hemi-area obtained from the binarized images (**i**, **j**). SCP: superficial capillary plexus, DCP: deep capillary plexus, OCTA: optical coherence tomography angiography

Moreover, for analysis of the hemi-VD disparities between the affected and unaffected areas in the SCP and DCP images were divided by a horizontal line into the superior and inferior region. The hemi-VD values were measured by binarization of the 320 × 160-pixel area (3 × 1.5 mm) and the hemi-VD disparity was calculated by subtracting the VD values of the affected hemi-area from the unaffected hemi-area (Fig. 4).

### Statistical analysis

The SAS program (version 9.4, SAS Institute Inc. Cary, North Carolina, USA) was used for statistical analysis; a *p*-value of < 0.05 was considered statistically significant. The values measured by two examiners were considered reliable if the intraclass correlation coefficient was ≥0.8. The mean of SSI values derived from *en face* OCTA images showed no significant differences between the two groups. (59.05 vs 60.62, *p* = 0.32).

The superficial and deep FAZ areas, average VD, and hemi-VD disparity in SCP and DCP were compared between the two groups using the Wilcoxon rank-sum test. Superficial and deep perifoveal capillary ring losses were analyzed using the Chi-square test, while the extent of capillary ring loss was analyzed using the Wilcoxon rank-sum test. Subgroup analyses based on the type of BRVO for perifoveal capillary network morphology and macular capillary VD were also performed using the Wilcoxon rank-sum test.

## Results

This study included a total of 43 patients: 21 patients with no ME recurrence and 22 patients with ME recurrence following intravitreal anti-VEGF injection therapy for BRVO-induced ME. The demographics and clinical characteristics of the eyes in the two groups are summarized in Table 1.

**Table 1:** Demographics and clinical characteristics of the study eyes

**Characteristics**	**no ME recurrence group (*n*** **= 21)**	**ME recurrence group (*n*** **= 22)**	***p*-value**
Mean age (year)	60.29 ± 14.26	64.00 ± 9.08	0.318^*^
Male /Female (no.)	8/13	7/15	0.667^†^
Right eye/Left eye (no.)	9/12	9/13	0.897^†^
Occlusion site, no. (%)			0.650^†^
Major BRVO	10 (47.6%)	12 (54.5%)	
Macular BRVO	11 (52.4%)	10 (45.5%)	
Presence of macular hemorrhage, no. (%)	14 (66.7%)	16 (72.7%)	0.665^†^
Mean logMAR BCVA at first visit	0.54 ± 0.45	0.61 ± 0.37	0.371^*^
Mean logMAR BCVA at 6 months	0.17 ± 0.21	0.33 ± 0.31	0.037^*^
Mean CMT before treatment	494.43 ± 164.62 um	550.82 ± 161.31 um	0.170^*^
Mean CMT at ME resolution	201.38 ± 32.17 um	194.05 ± 38.04 um	0.502^*^
Mean time to ME resolution, months	2.87 ± 2.16	2.30 ± 1.63	0.313^*^
Mean number of intravitreal injections	2.05 ± 1.12	2.86 ± 1.32	0.028^*^
Mean follow-up period, months (range)	8.38 ± 3.14 (6–15)	11.09 ± 4.82 (6–21)	0.036^*^

Values are presented as mean ± standard deviation unless otherwise indicated

*ME* Macular edema, *BRVO* Branch retinal vein occlusion, *logMAR* Logarithm of the minimum angle of resolution, *BCVA* Best corrected visual acuity, *CMT* Central macular thickness

* Statistics by Wilcoxon rank-sum test; † Statistics by Chi-square test

There were no statistically significant differences between the ME recurrence and no ME recurrence groups in terms of the mean ages (*p* = 0.318), sex (*p* = 0.667), eye involved (right or left; *p* = 0.897), location of vein occlusion (*p* = 0.650), presence of macular hemorrhage (*p* = 0.665), mean BCVA (logMAR) at the first visit (0.54 ± 0.45 vs. 0.61 ± 0.37, *p* = 0.371), mean CMT before treatment (494.43 ± 164.62 um vs. 550.82 ± 161.31um, *p* = 0.170), mean CMT after resolution of ME (201.38 ± 32.17um vs. 194.05 ± 38.04um, *p* = 0.502), and the mean duration until resolution of ME (2.87 ± 2.16 vs. 2.30 ± 1.63, *p* = 0.313).

However, the improvement in BCVA at 6 months after resolution of ME was significantly better in the no ME recurrence group than in the ME recurrence group (0.17 ± 0.21 vs. 0.33 ± 0.31, *p* = 0.037), while the mean number of injections until resolution of ME was higher in the recurrence group, (2.05 ± 1.12 vs. 2.86 ± 1.32, *p* = 0.028); this showed that the recurrence group required additional injections to achieve ME resolution.

Changes in the morphology of the perifoveal capillary network (FAZ area, perifoveal capillary ring) are summarized in Table 2. The mean FAZ area in the SCP (0.40 ± 0.11 mm^2^ vs. 0.52 ± 0.25 mm^2^, *p* = 0.035) and the DCP (0.48 ± 0.12 mm^2^ vs. 0.60 ± 0.26 mm^2^, *p* = 0.063) areas was wider in the ME recurrence group than in the no ME recurrence group. With respect to the intactness of the perifoveal capillary ring, the recurrence group showed a significantly higher number of cases with perifoveal capillary ring loss in the SCP (12/21 vs. 6/22, *p* = 0.047) and DCP (11/21 vs. 2/22, *p* = 0.002). The mean extents of perifoveal capillary ring loss in the SCP (17.3° vs. 69.5°, *p* = 0.006) and DCP (30.3 ° vs. 87.3°, *p* = 0.001) were greater in the ME recurrence group than in the no ME recurrence group.

**Table 2:** Perifoveal capillary network morphology on *en face* swept-source optical coherence tomography angiography images

**OCT angiography parameters**	**no ME recurrence** **group (*n*** **= 21)**	**ME recurrence group (*n*** **= 22)**	***p*-value**
**Superficial capillary plexus**			
Average FAZ area	0.40 ± 0.11 mm^2^	0.52 ± 0.26 mm^2^	0.035^*^
Intact perifoveal capillary ring, no. (%)	12 (57.1%)	6 (27.3%)	0.047^†^
Extent of the destructed capillary ring, no. (%)			
1 clock hour	6 (28.6%)	3 (13.6%)	
2 clock hours	3 (14.3%)	5 (22.7%)	
3 clock hours	0 (0.0%)	2 (9.1%)	
4 clock hours	0 (0.0%)	2 (9.1%)	
5 clock hours	0 (0.0%)	0 (0.0%)	
6 clock hours	0 (0.0%)	4 (18.2%)	
Average extent of capillary ring loss, °	17.3	69.5	0.006^*^
**Deep capillary plexus**			
Average FAZ area	0.48 ± 0.12 mm^2^	0.60 ± 0.26 mm^2^	0.063^*^
Intact perifoveal capillary ring, no. (%)	11 (52.4%)	2 (9.1%)	0.002^†^
Extent of the destructed capillary ring, no. (%)			
1 clock hour	5 (23.8%)	5 (22.7%)	
2 clock hours	2 (9.5%)	2 (9.1%)	
3 clock hours	2 (9.5%)	4 (18.2%)	
4 clock hours	0 (0.0%)	5 (22.7%)	
5 clock hours	0 (0.0%)	1 (4.6%)	
6 clock hours	1 (4.8%)	3 (13.6%)	
Average extent of capillary ring loss, °	30.0	87.3	0.001^*^

Values are presented as mean ± standard deviation unless otherwise indicated

*OCT* Optical coherence tomography, *ME* Macular edema, *FAZ* Foveal avascular zone

* Statistics by Wilcoxon rank-sum test; † Statistics by Chi-square test

The mean VD in the SCP (76.70 ± 9.06 vs. 78.07 ± 7.66, *p* = 0.298) and the DCP (73.71 ± 7.39 vs. 71.87 ± 6.22, *p* = 0.190) showed no significant differences between the two groups. However, the hemi-VD disparity between the unaffected and affected areas in the SCP (9.26 ± 10.68 vs. 13.08 ± 8.81, *p* = 0.031) and DCP (14.10 ± 12.42 vs. 21.73 ± 12.10, *p* = 0.017) showed statistically significant differences between the groups (Table 3).

**Table 3:** Macular capillary density on *en face* swept-source optical coherence tomography angiography images

**OCT angiography parameters**	**no ME recurrence group (*n* = 21)**	**ME recurrence** **group (*n* = 22)**	*p*-value^*^
**Superficial capillary plexus**			
Macular vessel density (3 × 3-mm area)	76.70 ± 9.06	78.07 ± 7.66	0.298
Hemi-vessel density (3 × 1.5-mm area)			
Hemi-vessel density in the unaffected area	81.49 ± 9.82	84.40 ± 9.72	0.215
Hemi-vessel density in the affected area	72.23 ± 16.62	71.32 ± 9.87	0.544
Hemi-vessel density disparity	9.26 ± 10.68	13.08 ± 8.81	0.031
**Deep capillary plexus**			
Macular vessel density (3 × 3-mm area)	73.71 ± 7.39	71.87 ± 6.22	0.190
Hemi-vessel density (3 × 1.5-mm area)			
Hemi-vessel density in the unaffected area	81.68 ± 7.70	82.55 ± 9.20	0.512
Hemi-vessel density in the affected area	65.57 ± 9.50	68.82 ± 8.38	0.040
Hemi-vessel density disparity	14.10 ± 12.42	21.73 ± 12.10	0.017

Values are presented as mean ± standard deviation unless otherwise indicated

*OCT* Optical coherence tomography, *ME* Macular edema

*Statistics by Wilcoxon rank-sum test

The values about the macular capillary morphology and macular VD based on the type of BRVO between two groups are presented in Table 4. There were no statistically significant differences between the major BRVO and macular BRVO in both the ME recurrence group and the no ME recurrence group with regard to the mean FAZ area, the mean extent of perifoveal capillary ring loss, the mean VD, the hemi-VD disparity. In patients with major BRVO, the ME recurrence group showed statistically significant values of the mean FAZ area in the SCP (*p* = 0.022), the mean extent of perifoveal capillary ring loss in the DCP (*p* = 0.006), hemi-VD disparity in the SCP (*p* = 0.022) than the no ME recurrence group. In patients with macular BRVO, the ME recurrence group showed statistically significant values of the mean FAZ area in the DCP (*p* = 0.049), the mean extent of perifoveal capillary ring loss in the SCP and DCP (*p* = 0.007, *p* = 0.015), the hemi-VD disparity in the DCP (*p* = 0.026) than the no ME recurrence group.

**Table 4:** Subgroup analysis based on the occlusion type for foveal vascular zone area, capillary ring loss, vessel density and hemi-vessel density disparity

	**no ME recurrence group**		**ME recurrence group**				***p***_***1***_-value^*^ (between A and C)	***p***_***2***_-value^*^ (between B and D)
	**A:major BRVO (*n*** **= 10)**	**B:macular BRVO (*n*** **= 11)**	*p*-value^*^	**C:major BRVO (*n*** **= 12)**	**D: macular BRVO (*n* = 11)**	*p*-value^*^		
**Superficial capillary plexus**								
**Mean FAZ area (mm**^**2**^**)**	0.40 ± 0.10	0.39 ± 0.11	0.484	0.50 ± 0.14	0.56 ± 0.37	0.312	0.022	0.123
**Mean extent of ring loss (°)**	21	13.5	0.274	72.5	66	0.472	0.078	0.007
**Macular VD**	78.21 ± 10.82	75.32 ± 7.39	0.960	77.22 ± 7.62	79.08 ± 7.98	0.264	0.334	0.154
**Hemi-VD disparity**	8.91 ± 12.55	9.81 ± 9.28	0.189	14.41 ± 10.72	11.47 ± 5.95	0.409	0.022	0.171
**Deep capillary plexus**								
**Mean FAZ area (mm**^**2**^**)**	0.47 ± 0.16	0.49 ± 0.11	0.374	0.55 ± 0.14	0.68 ± 0.37	0.245	0.098	0.049
**Mean extent of ring loss (°)**	15	43.5	0.097	85	90	0.448	0.006	0.015
**Macular VD**	73.84 ± 8.21	73.59 ± 6.97	0.154	70.21 ± 6.35	73.87 ± 5.71	0.089	0.111	0.484
**Hemi-VD disparity**	13.76 ± 13.22	14.42 ± 12.29	0.401	18.95 ± 10.31	25.06 ± 13.75	0.206	0.099	0.026

Values are presented as mean ± standard deviation unless otherwise indicated

*ME* Macular edema, *BRVO* Branch retinal vein occlusion, *FAZ* Foveal avascular zone, *VD* Vessel density

*Statistics by Wilcoxon rank-sum test

## Discussion

This retrospective study aimed to investigate how changes in the macular capillary morphology and the macular VD affect the recurrence of ME associated with BRVO. On *en face* OCTA images acquired after resolution of ME, the recurrence group showed a less intact perifoveal capillary ring and a broader range of ring loss than did the no ME recurrence group. Moreover, the recurrence group showed a larger hemi-VD disparity between the unaffected and affected areas in the SCP and DCP and a lower hemi-VD in the affected area in the DCP.

The most common causes of visual loss in BRVO cases are ME and macular ischemia [19, 20]. Although the definition of macular ischemia remains unclear, the occurrence of ME and that of macular ischemia are closely related [21, 22]. Sim et al. [21] defined macular ischemia as FAZ expansion, and non-perfusion in the perimacular capillaries. Meanwhile, Finkelstein [22] reported that macular ischemia is associated with the destruction of the perifoveal capillary ring, while Wakabayashi et al. [23] defined a capillary ring loss of ≥1/4 as FAZ destruction. In the present study, although a definition of macular ischemia was not defined, we analyzed whether the changes in the capillary network (the FAZ area, capillary ring morphology) and macular VD (3 × 3-mm macular region, the hemi-VD disparity) affected ME recurrence.

The mean superficial and deep FAZ areas in healthy people is 0.2–0.4 mm^2^ and 0.3–0.6 mm^2^, respectively [24–26]. Changes in the FAZ area and VD have been reported as important biomarkers for the progression of diabetic retinopathy and retinal vascular diseases and the prognosis of visual acuity [27–29]. These factors are more useful to OCTA than to fluorescein angiography (FAG) [30, 31].

Wakabayashi et al. [23] reported that smaller FAZ areas resulted in better visual acuity after resolution of ME, and Parodi et al. [26] reported that the association between ME and visual loss was weak, although an increase in the FAZ area was closely associated with visual loss. As shown, most of the previous studies reported associations between the FAZ area and the visual prognosis in cases of retinal vascular disease [17, 23, 26–29]. However, studies on the association between the FAZ area and the ME recurrence are lacking. Our study found that the recurrence group had wider superficial and deep FAZ areas after resolution of ME than did the no ME recurrence group, with the values for the superficial FAZ area showing greater statistical significance. Unfortunately, we did not analyze whether the FAZ areas changes over time after ME resolution; therefore, additional studies are needed to establish the changes in the FAZ areas following ME resolution.

In the present study, the extent of perifoveal capillary ring loss in the SCP and DCP was significantly higher in the recurrence group than in the no ME recurrence group, and it indicated that ring destruction was more severe at the DCP level than at the SCP level in the recurrence group. Identification of the DCP status by OCTA is very important for the detection of macular ischemia [32]. Deep capillaries play a role as a watershed zone that supplies blood to the outer plexiform and inner nuclear layers [24], and the necessary oxygen to the inner segments of visual cells under scotopic conditions [33]. Destruction of a capillary ring is an indicator of macular ischemia, and destruction of the superficial and deep perifoveal capillary rings promotes VEGF secretion, which may cause ME to occur more readily because of increased vascular permeability.

The relationship between retinal perfusion state and ME recurrence in BRVO has not been established. Hasegawa and colleagues [34] reported that patients with a severe reduction in the macular VD on *en face* OCTA SCP images exhibited fewer recurrences of edema and required fewer intravitreal anti-VEGF injections. Sakimoto and colleagues [35] divided the macular perfusion into three grades using FAG; full perfusion area, partial perfusion area, and nonperfusion area. They found that a partial perfusion area with a dilated and irregular capillary net was a source of macular edema.

The macular VD is the numerical value of the area occupied by retinal large vessels and capillary networks in binary reconstructed images. It is widely used to evaluate retinal microvascular state quantitatively. In this study, we checked the macular VD using 3 × 3 mm scan patterns and the hemi-VD disparity of both the SCP and DCP. Our results showed no difference in the macular VD between the recurrence and no ME recurrence groups, although the recurrence group showed greater hemi-VD disparity between the affected hemi- area and the unaffected hemi-area in the SCP and DCP. Moreover, the hemi-VD disparity in the DCP was particularly higher in the ME recurrence group.

The possible explanations for the opposite outcomes between macular VD versus hemi-VD disparity and edema recurrence are as follows. The VD does not provide retinal vascular flow states such as flow rates and leaks that evaluate the activation of retinal vein occlusion. The hemi-VD disparity can reflects the difference of ischemic damage from retinal vein occlusion in the affected areas and the unaffected areas. In addition, the secretion of VEGF is stimulated by the relatively hypoxic retina, especially the border zone of the nonperfusion area. We suggest that the amount of VEGF secreted by retinal tissues increases with the severity of the hemi-VD disparity. Rather than the macular VD, the VD disparity in hemi-areas may reflect disturbances in edema control and contribute to the recurrence of ME in BRVO. Yeung et al. [36] found that the ratio of VD in the DCP relative to the SCP is more important in regulating the macular fluid dynamics. The capillary damages in the DCP may affect the macular edema. Our study showed that the hemi-VD disparity in the DCP was higher in the ME recurrence group, which is more related to ME recurrence in BRVO.

Hayreh et al. [20] reported that the mean time to resolution of ME was similar in cases of major and macular BRVO, while visual improvement was worse in cases of major BRVO. However, there was no mention of the association between the location of the occlusion and ME recurrence. In the present study, we divided the two types according to the location of vein occlusion and analyzed the relationship between changes in macular capillary networks of two subgroups (major BRVO and macular BRVO) and ME recurrence. We found no statistically significant differences between the major type and the macular type of BRVO in the ME recurrence and no ME recurrence groups. As shown Table 4, ME recurrence group showed a wider FAZ area, a greater capillary ring loss and a larger hemi-VD disparity than no ME recurrence group regardless of the occlusion type.

This study has some limitations. Because the images were acquired from just a single round of OCTA, a relatively small number of samples were analyzed after the exclusion of poor-quality images. Furthermore, because of limitations in the built-in OCTA analysis algorithm, we calculated the FAZ area by manually drawing the inner boundaries and analyzed the VD in the macular capillary network using the ImageJ software. The new validated algorithm will enhance the results of future studies. Finally, we did not identify the extent of the retinal nonperfusion area by using FAG, while VD was only measured in a 3 × 3-mm macular region on OCTA images. Therefore, future studies should use wide-viewing OCTA images to examine the change in the macular capillary network morphology over time after ME resolution.

## Conclusions

In conclusion, perifoveal capillary ring destructions and hemi-VD disparities could be related to the recurrence of ME in patients with BRVO. The greater the destruction of the foveal capillary ring, the greater the hemi-VD disparity and the lower the hemi-VD in the areas affected by BRVO, these factors may be correlated with a higher risk of ME recurrence.

## Data Availability

The datasets used and/or analyzed during the current study are available from the corresponding author on reasonable request.

## Abbreviations

BRVO: Branch retinal vein occlusion
CMT: Central macular thickness
DCP: Deep capillary plexus
FAG: Fluorescein angiography
FAZ: Foveal avascular zone
ME: Macular edema
OCT: Optical coherence tomography
OCTA: Optical coherence tomography angiography
SCP: Superficial capillary plexus
SSI: Signal strength intensity
SS-OCT: Swept-source optical coherence tomography
VD: Vessel density
VEGF: Vascular endothelial growth factor
